# The design and methodology of a usability protocol for the management of medications by families for aging older adults

**DOI:** 10.1186/s12911-019-0907-8

**Published:** 2019-09-05

**Authors:** Y. Quintana, D. Fahy, A. M. Abdelfattah, J. Henao, C. Safran

**Affiliations:** 1 Harvard Medical School, Boston, MA USA; 2 Division of Clinical Informatics, Department of Medicine, Beth Israel Deaconess Medical Center, Boston, MA USA; 3 Harvard T.H. Chan School of Public Health, Boston, MA USA

**Keywords:** mHealth, Usability, Older adults, Health information technology, User interface design

## Abstract

**Background:**

Health research apps often do not focus on usability as a design priority. This is problematic when the population of interest is disproportionately underrepresented as users of mobile apps, especially observed with aging older adults (> = 75). Challenges with the adoption of health information technology (HIT) among this group are exacerbated by poor design and user interface/experience (UI/UX) choices. This protocol describes the testing and evaluation process of one HIT app for the family-based collaboration platform InfoSAGE.

**Methods:**

We aim to recruit twenty subjects from both informal family-caregivers and aging older adults to examine the usability of the InfoSAGE mobile medication manager. Participants will be audio and visually recorded, in addition to the use of screen capture recordings, while ‘thinking aloud’ as they complete eight common use-case scenarios. Multiple independent reviewers will code video and audio recordings for thematic analysis and use problems will be evaluated. Success and failure of each scenario will be determined by completion of sub-events. Time-to-complete analysis will be used to ascertain the learning curve associated with the app.

**Discussion:**

Frequently observed problem areas will be used as the basis of further evolution of the app, and will further inform generalized recommendations for the design of HIT apps for research and public use. This study aims to improve the model of development for dual user populations with dissimilar technological literacy to improve retention and use. Results of this study will form the foundation of a design framework for mobile health apps.

## Background

Longevity is increasing worldwide [1], and in the United States the ‘baby-boomer’ generation is rapidly approaching the age of ballooning medical costs that results from higher utilization due to age-related morbidities [2–4]. The challenges associated with these coming stressors will require scalable solutions throughout the healthcare spectrum. One area of promise is in the use of mobile health information (mHealth) technology to bring efficient, cost-effective, care to a wide audience [5, 6]. While elders are increasing using more mobile phones [7–9], challenges remain in adoption and technical literacy, especially as age rises [10–12]. The digital-divide has been decreasing in the last ten years, but is a scarcity of systematically designed studies for the evaluation of the usability of mHealth solutions in mixed-age populations, aimed at both informal caregivers and aging older adults.

Many studies have shown the potential negative impact of poorly designed information technology on facilitating medical errors [13, 14], and specific problems regarding regarding usability of mobile apps for medication [6, 15]. Usability testing has been applied in the assessment of health information system safety to identify and prevent medical errors and patient safety risks that may arise from the use of health information systems. Specifically, such methods have begun to be applied to the assessment of the impact of user interface features and design choices on medical error [16]. One study [6] evaluated a prototype app that simulates medication tracking using an iPad and found that users struggled with screen glare, button activation, and the “drag and drop” function [6], which makes it difficult for the significant number of users with poor vision to correctly use those apps - an estimated 1 in 5 North American adults aged 75 or over have a self-reported “seeing diability” [17]. Low health literacy is another barrier to effective interaction with technology - about one-half of North American adults have low literacy, meaning they lack the literacy skills needed for everyday life, [18] and between 46 and 60% also have low health literacy and struggle to “obtain process, and understand basic health information and services needed to make appropriate health decisions” [19, 20].

Usability problems in mobile apps lead to reduced utilization, lower rates of user retention, and increased user frustration [21] users who have difficulties navigating an app, understanding button configuration or layout, or find features to be too convoluted or complex are less likely to continue use [21, 22].

**Fig. 1:**
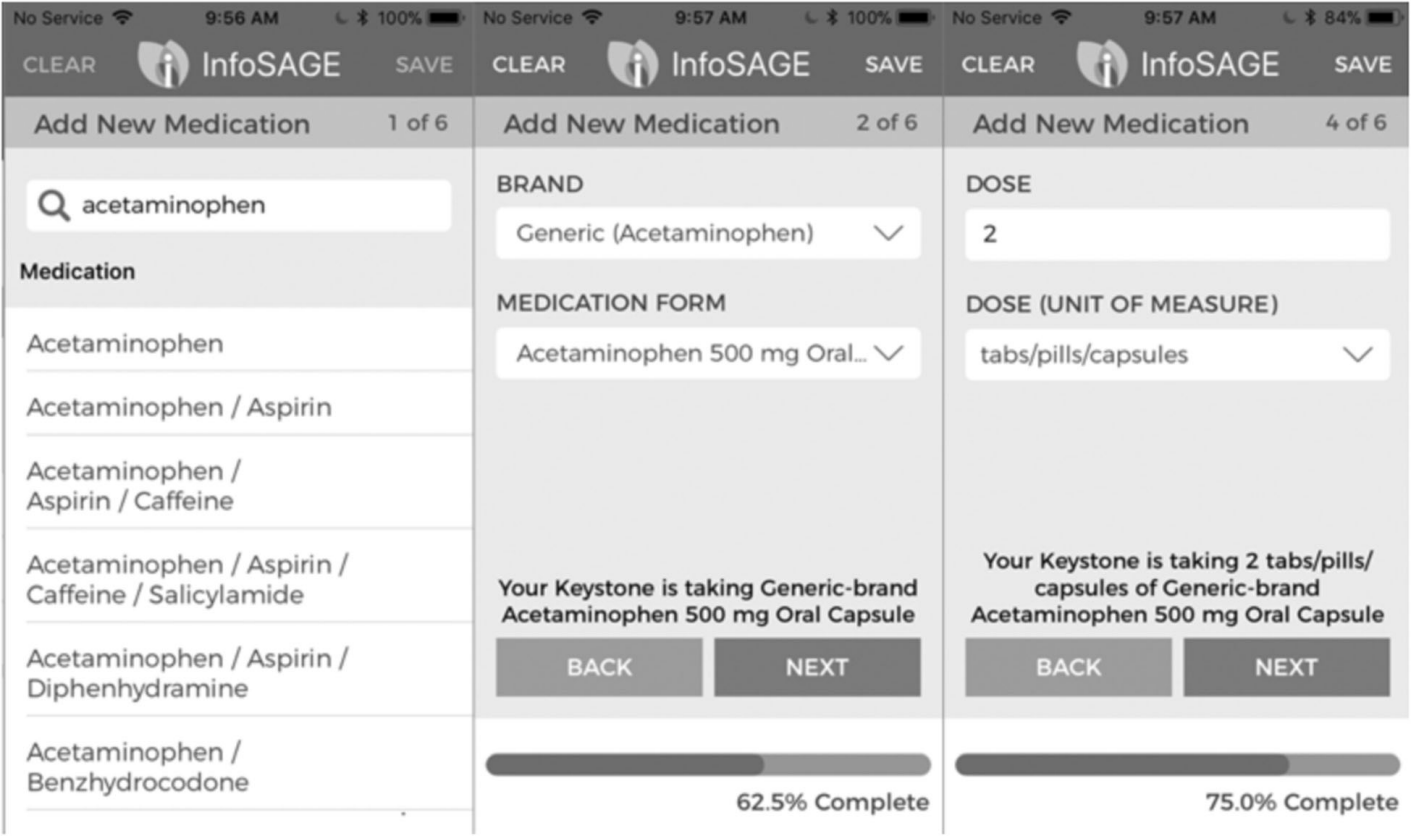
Sample medication addition using the infosage medication manager

The InfoSAGE platform is a free, mobile and web-based application providing features and tools for the informal caregiving of aging older adults through a shared family network [5]. One of the primary tools of InfoSAGE is the mobile medication manager (Fig. 1), which enables collection of prescription and over-the-counter medications and facilitates documentation of current and discontinued medications, pill images, dosages, and permits scheduling reminders [5]. Although the app is free to use and published publically, there has not been a formal evaluation of the usability within the dual populations serviced by InfoSAGE to date.

To these aims, this protocol describes a systematic method of evaluating a mobile medication management system within the InfoSAGE platform, usable on both iOS and Android operating systems. While the specifics are focused on the InfoSAGE app, the method and approach should be widely applicable to any app under evaluation for age-appropriate usability.

## Methods/design

### Research objective

The result of this study will be an evaluation of common problems encountered by frail older adults and their informal caregivers using this mobile app, and a set of recommendations for the design of mobile apps for elders based on our observations and analysis.

### Ethical approval and recruitment

The study is approved for ethical review by the Beth Israel Deaconess Medical Center Institutional Review board. Recruitment is on-going, beginning in April 2018, with a target convenience sample of 10 informal caregivers and 10 frail older adults drawn from local advertising, flyers, and online message boards, and a word-of-mouth and grassroots approach through collaborating partners.

### Procedure

Demographic and baseline comfort with the Internet, technology, and apps will be gathered prior to testing. After completion of the test scenarios, participants will be asked to complete a modified standard usability survey on a Likert scale and will have the opportunity to give open-ended feedback on the testing process, the app, and the scenarios (Table 1). Acceptability of use will be evaluated from the responses to the surveys.

**Table 1:** Post-testing surveys and open-ended responses

Post-Testing Survey - Likert Scale (1–7)	
Overall, I am satisfied with the ease of completing these tasks	
Overall, I am satisfied with the amount of time it took me to complete these tasks	
Overall, I am satisfied with the usefulness of the on-line help for completing these tasks	
How would you rate the difficulty of completing the task scenarios?	
Overall, after completing these tasks, I feel that this could potentially be used on a regular basis as part of my patient care, or my care recipient’s care, and for communicating my current list of medications with my care provider.	
Post-Testing Interview	
Did you understand the tasks that you were asked to complete?	
Are there any tasks that you found particularly difficult to use?	
What changes would you suggest to make the system easier to use?	
Is there anything about this usability test process or tasks scenarios that can be improved?	
Do you feel that this medication system could potentially improve your patient care?	

Testing will take place in a controlled office environment, using supplied development iPads using the publicly available InfoSAGE app. Audio and video recording will be utilized, but video recording will be limited to hands only. Participants will be asked to ‘think aloud’ while interacting with the app during testing scenarios. No faces or other identifiers will be recorded. During testing, touches and actions performed on the development iPad will be captured by screen recording software. Participants will be supplied with a test account, with full logging enabled to remove potential problems in registration or log ins. Prior to testing, participants will be given a brief overview of the InfoSAGE platform, the tiered access system, and a brief overview of its main features. Participants will not be instructed in how to use the app and will only receive help if they are unable to continue with the testing process. Any help provided will be noted.

**Fig. 2:**
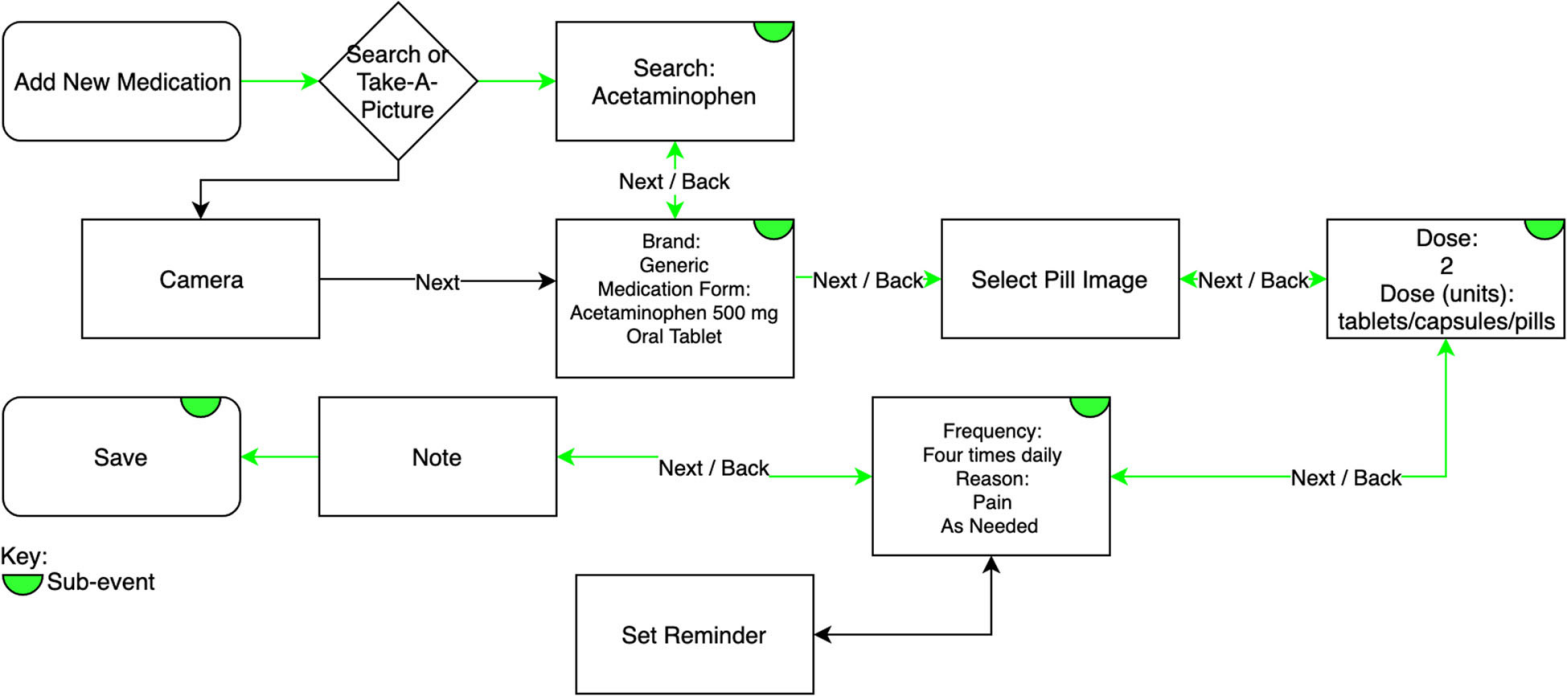
Scenario 3, addition of acetaminophen, optimal path flowchart and subevent

Eight individual scenarios were developed based on use cases frequently observed on InfoSAGE and were modified through internal testing with naïve users (Table 2). Each scenario is divided into subevents, or check-points, for further granularity of evaluation. Any staff help received will be evaluated in comparison to these subevents and if the help is deemed instrumental the subevent will be marked as failed. All subevent must be completed for the entire scenario to be considered passed. Scenarios one and two are designed to be used as a gauge for rapidity of familiarity, differing only in medication name. Scenarios four, five, seven, and eight are designed to evaluate the navigation, tactility and location of navigation elements (buttons, switches, links), and descriptive language used. Scenarios three (Fig. 2) and seven use more advanced medication entry elements and will be used to evaluate technological and health literacy.

**Table 2:** Scenario tasks and sub-events

Scenario	Scenario Description	# Sub-events
1	Add Lisinopril 5 mg, 1 tablet once daily	4
2	Add Warfarin 5 mg, 1 tablet once daily	4
3	Add Acetaminophen, 500 mg tablet, 2 tablets by mouth every 6 h, as needed for pain	7
4	View the side-effects of Lisinopril	2
5	Review drug-drug interactions	1
6	Modify the dose of Warfarin to 7.5 mg once daily	3
7	Inactivate Acetaminophen	1
8	Email medication list	1

### Evaluation and analysis

Audio transcripts will be transcribed and coded for broad themes. A heuristic method of coding will be performed on the video recording and screen capture. Two analysts will individually evaluate the videos, using a shared code dictionary, noting the events and codes through the Behavioral Observation Research Interactive Software (BORIS, v.7) [23]. Review of the coded videos will be conducted by the entire team. Initial cases will be used to further develop the analysis methodology, with codes undergoing assessment for applicability and appropriateness. This method will ensure the relevance and maximize the value of the categorizing and coding.

Each case will be reviewed by the team, and differences in codes and events noted. Although this is a qualitative study and we expect to have differences in coding between analysts, we will compare each event and code against the code definition for appropriateness. Incorrectly applied codes will be adjudicated as a group. Broadly, we expect the codes to fall into the following categories: data display visibility issues, navigation problems, data entry problems, content comprehension, attention problems or other cognitive confusion, and issues of health literacy. A qualitative assessment will be completed on the problems users had with cognitive confusion based on the verbal comments made by the test subjects.

Interrater correlation will be evaluated by a two-way, intraclass correlation coefficient [24]. Individual codes will be thematically grouped and evaluated by scenario and subject. Additionally, time-series visualization will be used to compare clustering of all subjects’ aggregated events to identify commonly observed navigation or cognitive problems. Finally, intraclass correlation will be determined between coders for failure/success of each scenario.

Secondary to the evaluation of common problems identified, we will also examine the uptake of learning by comparing the differences in time to complete for scenarios one and two in aggregate, and by comparing the time to a group of familiar, expert users. Additionally, navigation errors, hesitation, and comments or observations of frustration and annoyance will be quantified between the two scenarios and analyzed as a condition of learning.

Post-testing feedback from participants will be used to inform future design decisions and will comments will be assessed along with observations and quantitative metrics to modify navigation flow and user interface/user experience. Demographics, Internet comfort-level and app expertise will be examined for correlation to age and tech literacy of the participants.

## Discussion

We aim to produce a generalizable set of recommendations for the future design of mHealth apps targeted towards mixed age populations of informal caregivers and their aging older adults. Through design and iteration, we hope to form the basis for a framework to support further usability testing of mobile health applications.

## Data Availability

Not applicable. Our manuscript does not contain any data.

## Abbreviations

BORIS: Behavioral Observation Research Interactive Software
HIT: Health Information Technology
InfoSAGE: Information Sharing Across Generations and Environments
UI: User Interface
UX: User Experience
